# A draft chromosome-scale genome assembly of a commercial sugarcane

**DOI:** 10.1038/s41598-022-24823-0

**Published:** 2022-11-28

**Authors:** Jeremy R. Shearman, Wirulda Pootakham, Chutima Sonthirod, Chaiwat Naktang, Thippawan Yoocha, Duangjai Sangsrakru, Nukoon Jomchai, Sissades Tongsima, Jittima Piriyapongsa, Chumpol Ngamphiw, Nanchaya Wanasen, Kittipat Ukoskit, Prapat Punpee, Peeraya Klomsa-ard, Klanarong Sriroth, Jisen Zhang, Xingtan Zhang, Ray Ming, Somvong Tragoonrung, Sithichoke Tangphatsornruang

**Affiliations:** 1 National Omics Center, National Science and Technology Development Agency, Pathum Thani, Thailand; 2 National Biobank of Thailand, National Science and Technology Development Agency, Pathum Thani, Thailand; 3 National Center for Genetic Engineering and Biotechnology, National Science and Technology Development Agency, Pathum Thani, Thailand; 4 Department of Biotechnology, Faculty of Science and Technology, Thammasat University, Rangsit Campus, Klong Luang, Pathum Thani Thailand; 5 Crop Production, Mitr Phol Innovation and Research Center, Pathum Thani, Thailand; 6 Center for Genomics and Biotechnology, Fujian Agriculture and Forestry University, Fuzhou, Fujian China

**Keywords:** Agricultural genetics, Evolutionary biology, Genome

## Abstract

Sugarcane accounts for a large portion of the worlds sugar production. Modern commercial cultivars are complex hybrids of *S. officinarum*, *S. spontaneum*, and several other *Saccharum* species, resulting in an auto-allopolyploid with 8–12 copies of each chromosome. The current genome assembly gold standard is to generate a long read assembly followed by chromatin conformation capture sequencing to scaffold. We used the PacBio RSII and chromatin conformation capture sequencing to sequence and assemble the genome of a South East Asian commercial sugarcane cultivar, known as Khon Kaen 3. The Khon Kaen 3 genome assembled into 104,477 contigs totalling 7 Gb, which scaffolded into 56 pseudochromosomes containing 5.2 Gb of sequence. Genome annotation produced 242,406 genes from 30,927 orthogroups. Aligning the Khon Kaen 3 genome sequence to *S. officinarum* and *S. spontaneum* revealed a high level of apparent recombination, indicating a chimeric assembly. This assembly error is explained by high nucleotide identity between *S. officinarum* and *S. spontaneum*, where 91.8% of *S. spontaneum* aligns to *S. officinarum* at 94% identity. Thus, the subgenomes of commercial sugarcane are so similar that using short reads to correct long PacBio reads produced chimeric long reads. Future attempts to sequence sugarcane must take this information into account.

## Introduction

Sugarcane is an important crop species and is the major source of processed sugar in the world. The name sugarcane does not refer to a single species, but rather refers to any of several species in the genus *Saccharum*. Taxonomic classification of these species is difficult, confounded by many years of cultivation and cross breeding, which makes phenotypic classification unreliable, and has been a topic of much debate. It was originally considered that six species exist: *S. spontaneum*, *S. robustum*, *S. officinarum*, *S. sinense*, *S. edule* and *S. barberi*^1^. Although, now it is common for *S. edule* to be excluded from this genus because in situ hybridisation revealed it to be a likely hybrid between *S. officinarum* and *S. robustum*, leaving only five species^1^. Only two of these species, *S. robustum* and *S. spontaneum*, are considered wild species, and the remaining species are all cultivated^2,3^.

Modern commercial cultivars of sugarcane are complex hybrids of *S. officinarum* as the maternal donor crossed with *S. spontaneum* and, to a lesser extent, some other species and hybrids (for review see^1,4,5^). Sugarcane is believed to have originated in the South Pacific, but was widely dispersed by early explorers making it difficult to pinpoint the exact origin. It is believed that *S. spontaneum* originates from India, but can be found growing wild from eastern and northern Africa, through the Middle East, to India, China, South East Asia, and through the Pacific to New Guinea. The other wild species, *S. robustum*, is indigenous to New Guinea and can be found along river banks. It is considered that *S. officinarum* also most likely originated in New Guinea and was likely derived from *S. robustum*. Modern commercial cultivars have complex polyploid genomes as a result of many generations of hybridisation.

The genome of modern commercial sugarcane consists of ten unique chromosomes with 8–12 copies of each, giving a variable chromosome count of 100–120^6–9^. The genome of *S. officinarum* consists of ten unique chromosomes and a copy number of eight, 2n = 8x = 80 (GCA_020631745.1). The genome of *S. spontaneum* consists of eight unique chromosomes and a copy number of eight, 2n = 8x = 64^10^. Some variability exists for *S. spontaneum* chromosome number with cases of 9 and 10 chromosomes identified^11,12^. When *S. officinarum* is crossed with *S. spontaneum* the offspring have two copies of the *S. officinarum* chromosomes and one copy of the *S. spontaneum* chromosomes^9^. Both the *S. officinarum* and *S*. *spontaneum* genomes have a high degree of gene collinearity to *S.orghum bicolor*^6,10,13,14^. A one haploid *S. spontaneum*, AP85-441 1n = 4x = 32, genome was sequenced and assembled into the full set of 32 chromosomes with each homoeologue separated into a distinct chromosome^10^. This *S*. *spontaneum* has strong gene collinearity to *S. bicolor*, despite having two fewer chromosomes, because the reduction in chromosome count was the result of fission in two chromosomes and translocation to the ends of four other chromosomes with little to no rearrangements^10^. The strong similarity between sugarcane and *S. bicolor* led many to suggest that that *S. bicolor* can be used as an aid in assembling the sugarcane genome and *S. bicolor* was often used as a reference for sugarcane transcriptome sequencing.

A monoploid mosaic sugarcane genome was assembled by generating and sequencing a series of bacterial artificial chromosomes that contained inserts of sugarcane R570 variety^15^. These sequences were then assembled into a single set of ten chromosomes aided by gene collinearity with *S. bicolor* to scaffold contigs. The result of such an approach was that each of the ten chromosomes were assembled, but the sequence of any particular locus can come from any of the homoeologous chromosomes, including potential mixing of chromosomes from the ancestral species *S. officinarum* and *S*. *spontaneum*^15^. This genome assembly is a valuable resource, but lacks much of the genetic variation that exists in the whole genome. Sequencing and comparing the nuclear and mitochondrial genomes of multiple sugarcane cultivars may yield some insight into the history of sugarcane and provide a valuable resource for genetic improvement.

The work presented here began as a collaboration effort to sequence and assemble *S. spontaneum*, *S. officinarum*, and a commercial cultivar, widely used in Thailand, known as Khon Kaen 3 (KK3). The intent was to use the genomes of the progenitor species, *S. spontaneum* and *S. officinarum*, to aid in assembly and annotation of the commercial variety KK3. All three were sequenced simultaneously using PacBio and the assembly, annotation and comparative analysis of KK3 is presented here.

## Materials and methods

### Sample and DNA/RNA extraction

The sugarcane we used is a popular commercial hybrid that has been developed in Thailand known as Khon Kaen 3 (KK3). This cultivar was generated by crossing K84-200 (ROC1 × CP63-588) with 85-2-352 (SP70-1143 × Q76) and is a cultivar that is commonly used in Southeast Asia. Leaf tissue was collected, with all relevant permissions obtained and in accordance with all relevant guidelines and legislation, from a single plant and used for DNA extraction with the standard CTAB method followed by clean-up using a DNeasy Mini spin column from Qiagen. Total RNA was extracted using the standard LiCl method from young leaf, mature leaf, meristem, young stem, and mature stem.

### Genome size estimation

The 2C DNA content was estimated by flow cytometry according to the protocol in Dolezel and Bartos^16^. Two reference species, *Zea mays* and *Pisum sativum*, were used for comparison.

### Genome sequencing and assembly

DNA was used to prepare libraries for the PacBio RSII following the Pacific Biosciences ‘Procedure and Checklist—20 Kb Template Preparation Using BluePippin Size-Selection System’ protocol. DNA (10 µg) was sheared with a Covaris gTube, 4500 rpm for 2 min and the BluePippin cassette used was ‘0.75% DF Marker S1 high-pass 15–20 Kb’ with selection of 12–50 Kb. Sequencing was performed for 700 cells on the PacBio RSII.

Raw reads longer than 10 Kb (27,848,780 reads totalling 428.2 Gb) were used as seed reads and reads shorter than 10 Kb were used to correct them using CANU v1.3^17^. The corrected long reads were then assembled using CANU v1.3 with an estimated genome size of 10 Gb. The assembly was then corrected using quiver. Default settings were used for each step as they are optimised for PacBio type data. Four Hi-C sequencing libraries were constructed following the steps described in Zhang et al.^10^ and sequenced on an Illumina HiSeq X Ten to generate 407,313,208 paired-end 150 bp reads. Hi-C was used to scaffold the assembled contigs into pseudo-chromosomes using the program ALLHiC, which is a version of HiRise software that was adapted for highly polyploid genomes^18^ and successfully used to assemble the *S. spontaneum* genome^10^. The ALLHiC parameters were derived from the data as described described in Zhang et al.^10^, with the exception that the k values were set to the expected number of haplotypes for KK3, even integers between 50 and 60 were tested, and the optimal k found to be 56.

### RNA-seq and annotation

Total RNA from stem (cane stalk), from the 9th internode, 3rd internode, 1st internode, mature leaf, and young leaf was collected from KK3 and immediately frozen in liquid nitrogen. Sequencing libraries were prepared from each collected tissue according to Ion Total RNA-Seq Kit ptocols and sequenced on an Ion S5 XL System.

Annotation of KK3 was performed by EvidenceModeler v1.1.1 r2015-07-03 using both transcriptome based evidence and ab initio prediction^19^. The transcriptome data consisted of EST data from the sugarcane SUCEST project^20,21^ and RNA-seq from KK3, this data was mapped to the genome assembly using PASA2 v2.0.1^19^. In addition, seven PacBio cells of RNA-seq was mapped to the genome assembly using GMAP^22^. The ab initio prediction program SNAP^23^ was trained using the PASA2 alignment assembly. All gene predictions were then combined by EvidenceModeler to generate consensus gene models using the following weights for each evidence type: PASA2 = 1; GMAP = 0.5; SNAP = 0.3.

### Repeat sequence annotation

RepeatModeler version 1.0.8 was used to identify and classify de novo repeat families on the assembled genome^24^. The repeat sequences identified in the genome were aligned to Genbank’s non-redundant protein database (using BLASTX with an E-value cutoff of 1 × 10^−6^) to exclude repeat sequences that contain large families of protein-coding genes. Repeat masking was performed on the assembled genome by RepeatMasker version 4.0.6 (http://repeatmasker.org) using repetitive sequences in the RepeastMasker consensus library (20150807; http://www.girinst.org) and the custom repeat library generated by RepeatModeler.

### Comparative genomics

The genome of *S. spontaneum* was aligned to the genome of *S. officinarum* using the NUCmer script of MUMmer3^25^. The coords output of NUCmer was analysed to determine the total amount of mapped sequence and the percent identity of the two genomes. Multiple overlapping alignments of individual sequences were reduce to a single range using the GenomicRanges package in R to calculate the total percent of *S. spontaneum* that mapped to *S. officinarum*. The same method was applied to the individual wheat subgenomes^26^ to determine identity statistics. See supplementary File 1 for a step-by-step description.

The KK3 genome assembly was compared to the genome assemblies of *S. officinarum* and *S. spontaneum* by mapping the KK3 contigs separately using the two progenitor assemblies as a reference. The mapping was performed to each reference using BWA-mem^27^ with default settings and the minimum match score (-T) set to 1000. The CIGAR string from the resulting SAM file was parsed to extract the ranges of each KK3 contig that could be mapped to each of the genomes. Cases where overlapping parts of the KK3 contigs mapped to multiple locations in the reference were reduce to a single range using the GenomicRanges package in R to calculate the percent of the KK3 assembly that mapped to each reference. Similarly, to calculate the percent of each reference that matched KK3, reference contigs with overlapping ranges of KK3 match were reduced to a single range. Mapping results using BWA-mem with default settings (more stringent) was used to differentiate between KK3 contigs that mapped equally to both references allowing contigs to be more accurately grouped. See supplementary File 1 for a step-by-step description.

## Results and discussion

### Sugarcane genome assembly

The KK3 genome was estimated to be 10.8 Gb (2C) based on flow cytometry. We assembled the KK3 genome using long PacBio reads into 104,477 contigs totalling 7 Gb. The contigs ranged in size from 10 kb to 1.8 Mb and had an N50 of 83 kb. Raw reads were mapped back to the contigs revealing a median read depth of 13. Based on the read depth of each contig compared to the median read depth, we estimated that approximately 4.6 Gb of sequence was single copy and 2.4 Gb was multiple copy that represent collapsed sequence. The KK3 contigs were compared to the assembled genomes of *S. officinarum* and *S. spontaneum* to identify which progenitor species they most resembled. This identified 4.9 Gb of the genome to have *S. officinarum* origins and 2.1 Gb with *S. spontaneum* origins. In addition, this comparison suggested that 6819 contigs showed recombination between the two ancestral genomes, which is a substantially larger than expected number of recombinations. We performed the same mapping using the R570 monoploid sugarcane genome^15^, and found 407 recombinant contigs. Accounting for differences in chromosome number, the KK3 assembly had three times the number of recombinant contigs than the R570 monoploid genome. The R570 monoploid genome is chimeric by design and this fact, coupled with the following results and discussion, led us to conclude that the KK3 genome assembly is artifactually chimeric.

**Figure 1:**
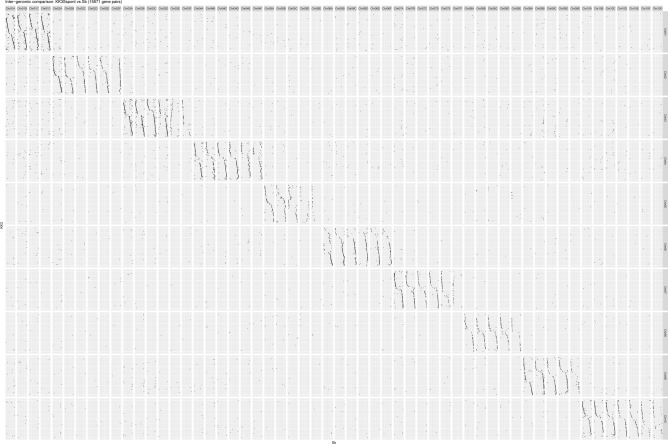
Gene synteny between the KK3 pseudo chromosomes and *Sorghum* *bicolor*. Dot plot showing the relative location of each homologous gene between the KK3 chromosomes and *Sorghum bicolor* chromosomes. Chromosomes are scaled to maintain relative distance between genes, but remove size differences between chromosomes. The chromosomes of KK3 are numbered according to which *S. bicolor* chromosome they represent and assigned a letter to differentiate between homoeologs.

**Figure 2:**
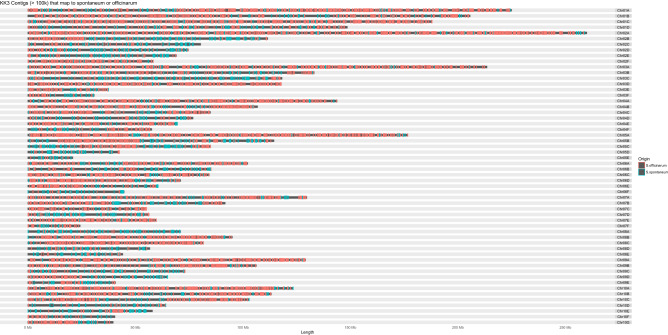
Chromosomes of KK3 showing length with similarity to each ancestral genome indicated by color. Each KK3 chromosome is represented by a horizontal bar with the length indicated on the x-axis. The ancestral origin of each contig larger than 100 kb, based on sequence alignment results, is indicated by colored rectangles on each chromosome. The size of colored rectangles is proportionate to the relative size of the contig, but not proportionate to the actual bp size. Pink represents contigs that had more sequence overlap with *S. officinarum* and teal represents *S. spontaneum*.

Scaffolding with Hi-C data produced 56 pseudo chromosomes containing 5.2 Gb, consistent with the 2C genome size estimation. There was 1.8 Gb of unplaced sequence, which consisted largely of alleleic sequence. Approximately 1.36 Gb (75%) of the unplaced sequence aligns to the pseudo chromosome scaffolds, leaving approximately 450 Mb of unplaced unique sequence. The unplaced sequence is mostly connected to allelic sequence, suggesting that it consists of prescence/absence alleles. Comparison of gene order and location in the 56 pseudo chromosomes to *Sorghum bicolor* was consistent with prior reports showing a high degree of gene colinearity (Fig. 1). However, all pseudo chromosomes contained sequence from both ancestral species (Fig. 2), which would require a large amount of recombination between the ancestral chromosomes. When only repeat masked sequence was analysed and plotted, the result looks the same (supplementary Fig. 1), meaning that the apparent recombination is not just repeat sequence or active transposon sequence. This was unexpected since Flourescence In Situ Hybridisation (FISH) and Genome In Situ Hybridisation (GISH) have shown that the genomes of each ancestral species remain largely isolated^7,9^. Genome in situ hybridisation has identified that 10–23% of the chromosomes of commercial sugarcane varieties are completely *S. spontaneum*^7,9^. However, when Cuadrado et al.^7^ used whole genome DNA from *S. officinarum* as one probe and whole genome DNA from *S. spontaneum* as another probe, they found that all chromosomes were labelled with the same probes because of high homology between the two species.

**Figure 3:**
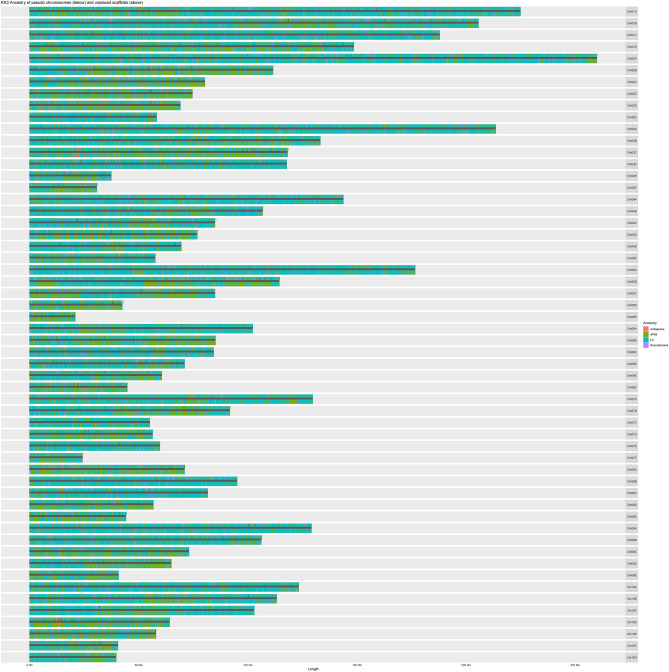
Chromosomes of KK3 showing two tracks per chromosome with the bottom track representing ancestry of scaffolded contigs and the top track showing ancestry of aligned unplaced contigs. Each KK3 chromosome is represented by a horizontal bar with the length indicated on the x-axis. The ancestral origin of each scaffolded contig, based on sequence alignment results, is indicated by colored rectangles on the bottom of each chromosome. The ancestral origin of each unplaced contig, based on sequence alignment results, is indicated by colored rectangles on the top of each chromosome. The size of colored rectangles is proportionate to the relative size of the contig, but not proportionate to the actual bp size. Teal represents contigs that had more sequence overlap with *S. officinarum* (LA), green represents *S. spontaneum* (AP85), pink represents contigs that mapped almost equally to both ancestors, and purple represents contigs that mapped half to one ancestor and half to the other.

We first considered that sequence similarity between the homoeologous chromosomes may have resulted in paired reads linking allelic sequence from separate homoeologous chromosomes. This could cause artefactual mixing of homoeologous sequence in the scaffolding step while maintaining the correct gene order. If this was the case, the unplaced allelic contigs would likely be from the opposite ancestral species as the corresponding pseudo chromosome sequence. However, this was largely not the case as 59% of the aligned unplaced contig sequence was identified as originating from the same ancestral species as the pseudo chromosome sequence to which it aligns (Fig. 3). We next partitioned the contigs based on ancestral origin and performed Hi-C scaffolding on each partition separately. We expected this would reconstruct each ancestral set of full chromosomes and partial segments of any recombinant chromosomes if that was the true sequence. The scaffolding of contigs identified as having *S. spontaneum* ancestry was attempted first, with the expected chromosome number set to 15. This produced 10 large scaffolds that ranged in size from 122 to 264 Mb and five small scaffolds that ranged in size from 24 to 38 Mb.

The *S. spontaneum* genome (n = 8) has gene synteny to *S. bicolor* (n = 10) with two of the *S. bicolor* chromosomes each split in half and fused onto the ends of other chromosomes giving rise to the chromosome count of 8^10^. Sequence data for *S. spontaneum* with n = 9 or n = 10 is not available, but similarly high gene synteny to *S. bicolor* would be expected as synteny is also found in *S. officinarum* and commercial cultivars. The obtained 10 large scaffolds could be consistent with ancestry of an *S. spontaneum* with n = 10. However, the gene order for this assembly relative to sorghum was substantially less colinear than the full assembly was, which means that partitioning the contigs in this way resulted in a lower quality assembly (Supplementary Fig. 2).

The amount of recombination observed in the KK3 assembly is much larger than would be expected, suggesting a problem with the assembly. The genome was assembled using only PacBio reads, which have a large error rate that is corrected by aligning short reads to the longest reads and correcting the long reads to generate consensus long reads^28^. If the progenitor genomes are similar enough, this error correction stage could result in reads from both subgenomes correcting long reads from both subgenomes and produce corrected long reads that are artificial chimeras of the two subgenomes. In such a situation, only the most divergent sequence between the two progenitors would produce accurate corrected long reads, while regions that are most conserved between the two would generate chimeric sequence skewing toward *S. officinarum* sequence. This process would explain the large amount of apparent recombination observed in the assembly and why there were so few *S. spontaneum* chromosomes found. This means that the homoeologous chromosomes are likely all chimeric and cannot be reliably used to dissect progenitor genome contribution. To investigate this possibility, the *S. spontaneum* genome was aligned to the recently assembled *S. officinarum* genome using NUCmer and it was found that 91.8% of the *S. spontaneum* genome aligned to the *S. officinarum* genome with an average nucleotide identity of 94.3%. This means that the error rate of the uncorrected PacBio reads (10–15% error rate)^28^ is higher than the percentage of difference between the two progenitor genomes and thus, the error correction step is probably producing chimeric reads rendering the entire assembly chimeric. The high amount of repeat sequence combined with the high ploidy also caused problems with the Hi-C paired sequence mapping (supplementary file 2) and may have generated scaffolds that contain homologous and homoeologous sequences, which would explain the large size variation within chromosome groups (Fig. 2).

The methods we used to assemble KK3 are essentially the methods that were used to successfully assemble the hexaploid wheat genome^26^. To investigate why it worked for wheat and not for sugarcane, we compared the individual subgenomes of hexaploid wheat to each other to add some perspective to the sugarcane progenitor genome similarity results. The wheat subgenomes are designated A, B and D^26^. We mapped the A genome against the B and D genomes and the B genome against the D genome. This showed that between 83.7 and 86.7% of each subgenome mapped to the other subgenomes at 91.2 to 91.4% identity, which is lower than the similarity between sugarcane progenitors, but still quite high. The wheat assembly project used short read sequencing to assemble contigs and PacBio reads to scaffold these contigs prior to using Hi-C to generate chromosome level scaffolds. Optical mapping of the wheat genome later identified the presence of some chimeric sequence and some sequence assigned to incorrect locations, which is likely to be, at least in part, caused by the high level of similarity between the subgenomes^29^. If a short read assembly method was used for KK3 sugarcane we would likely have a much larger number of correct shorter contigs. However, scaffolding using long reads would likely still produce chimeric scaffolds, similar to our current assembly. So the sugarcane genome may require an assembly approach where chromosomes are isolated and individually sequenced and assembled. Alternatively, updates to PacBio sequencing chemistry, known as HiFi reads (12–22 kb), occurred after the sequencing for this project was completed that sacrifice some read length for greatly increased accuracy, above 99%, and would avoid the chimeric long reads experienced in this work. However, the trade-off of shorter read length is considerably costly for a genome with such a high ploidy and repeat level and would result in reads not being able to span conserved allelic or repeat sequence, resulting in a more fragmented assembly. This means the high similarity of homoeologous chromosomes would likely still result in problematic allele mixing at repeat regions and regions of high sequence conservation. This would likely cause the generation of chimeric Hi-C scaffolded chromosomes, similar to a monoploid genome.

### Sugarcane genome annotation

The genome was annotated by combining protein data from the progenitor species, EST data from the SUCEST database^30^, RNA-seq data, and ab initio gene prediction methods. This resulted in 242,406 genes, which is a large number of genes, but this number is high because the assembly produced 4–7 copies of each chromosome with 6 copies being the most common. Grouping this gene set using Orthofinder produced 30,927 groups of orthologous genes. While most chromosomes had five or six homoeologous copies, very few orthogroups had genes from each homoeologous chromosome and the most common distribution was for each othogroup to include genes from two to four homoeologous chromosomes. Most orthogroups had multiple genes that occurred within a single homoeologous chromosome, which likely reflects the problems of the assembly. The genome was annotated to consist of 56.13% repeat sequence with the most prevalent repeat type being LTR Gypsy at 27.8%, followed by LTR Copia at 15.4% (Table 1). RepeatMasker masked 60.13% of the genome (stretches less than 10 bp between repeats are also masked) and 4238 of these masked regions were larger than 20 kb. This is likely an under-representation considering that the sequenced genome was 7 Gb and the measured genome was 10.8 Gb, meaning over 3 Gb of sequence is likely to be collapsed allele or repeat sequence.

**Table 1:** Repeat sequence annotated in KK3 genome.

Repeat class	No. of elements	Total bp masked	% genome masked
DNA/CMC-EnSpm	519,278	255,462,349	3.64
DNA/MULE-MuDR	240,450	77,168,806	1.10
DNA/PIF-Harbinger	721,693	153,386,043	2.19
LINE/L1	236,740	133,767,239	1.91
LTR/Copia	685,340	1,083,387,117	15.43
LTR/Gypsy	1,357,965	1,951,756,200	27.80
Unknown	743,186	286,426,659	4.08
Total	4,504,652	3,941,354,413	56.15

Of particular interest to sugarcane farming is the identification of genes that affect sugar production or provide resistance. There were 17,316 resistance genes that were annotated in the KK3 genome and 1077 genes that are involved in the starch and sucrose metabolism KEGG pathway. There were 294 sugar transporter genes identified in KK3, which is only slightly more than the 286 that were found in *S. spontaneum*. The number of genes for most transporter families was similar or reduced compared to *S. spontaneum*, with the polyol transporter (PLT) and tonoplast sugar transporter (TST) families showing a larger number of genes (Table 2). Considering the chimeric nature of the assembly it cannot be determined which progenitor genome provided which gene.

**Table 2:** Sugar transporter genes from *S. spontaneum* that were identified in KK3.

Transporter group	*S. spontaneum*	KK3
INT	15	12
pGlcT	8	6
PLT	66	102
SFP	22	18
STP	83	78
SUT	19	8
SWEET	53	38
TST	13	32
VGT	7	0
Total	286	294

## Conclusions

Assembling genomes from long read sequence data with scaffolding from Hi-C is the current gold standard in de-novo genome assembly and has produced chromosome level assemblies for a large number of species, including the hexaploid wheat genome. Application of this method to the auto-allopolyploid commercial sugarcane, KK3, appears to have produced a chimeric assembly because of a combination of consensus long read sequencing chemistry, the high ploidy level, and the high level of nucleotide identity between the genomes of the two progenitor species. Although the assembly produced from this work is unlikely to be a true reflection of what the actual genome should look like, it does at least capture much of the alleleic variation that exists in this commercial sugarcane and may be of some use when combined with one of the published monoploid mosaic sugarcane genomes or a future assembly that can overcome these problems. A good approach for sugarcane could be to isolate, sequence and assemble individual chromosomes. This work will hopefully benefit any future attempts to assemble difficult polyploid genomes, such as sugarcane.

## Supplementary Information

Supplementary Information 1.

Supplementary Information 2.

Supplementary Figure 1.

Supplementary Figure 2.

## Data Availability

All raw read data was deposited in the NCBI SRA data archive under the bioproject: PRJNA820955. The genome assemble can be found in the NCBI genome archive: JALQSO000000000.
