# Supplementary material for: A draft chromosome-scale genome assembly of a commercial sugarcane
Source: Sci Rep. 2022 Nov 28;12:20474. doi: 10.1038/s41598-022-24823-0 (PMC9705387; doi:10.1038/s41598-022-24823-0)
Supplement: Supplementary file 1 — Supplementary Information 1. [file 41598_2022_24823_MOESM1_ESM.docx]

# A draft chromosome-scale genome assembly of a commercial sugarcane

1. Jeremy R Shearman^1*^, Wirulda Pootakham^1^, Chutima Sonthirod^1^, Chaiwat Naktang^1^, Thippawan Yoocha^1^, Duangjai Sangsrakru^1^, Nukoon Jomchai^1^, Sissades Tongsima^2^, Jittima Piriyapongsa^2^, Chumpol Ngamphiw^2^, Nanchaya Wanasen^3^, Kittipat Ukoskit^4^, Prapat Punpee^5^, Peeraya Klomsa-ard^5^, Klanarong Sriroth^5^, Jisen Zhang^6^, Xingtan Zhang^6^, Ray Ming^6^, Somvong Tragoonrung^3^ & Sithichoke Tangphatsornruang^1*^
2. ^1^ National Omics Center, National Science and Technology Development Agency, Pathum Thani, Thailand.
3. ^2^ National Biobank of Thailand, National Science and Technology Development Agency, Pathum Thani, Thailand.
4. ^3^ National Center for Genetic Engineering and Biotechnology, National Science and Technology Development Agency, Pathum Thani, Thailand.
5. ^4^ Department of Biotechnology, Faculty of Science and Technology, Thammasart University, Rangsit Campus, Klong Luang, Pathumtani 12121, Thailand.
6. ^5^ Crop Production, Mitr Phol Innovation and Research Center, Pathumthani, 12120, Thailand.
7. ^6^ Center for Genomics and Biotechnology, Fujian Agriculture and Forestry University, Fuzhou, Fujian 350002, China.
8. * Corresponding authors

**Supplementary File 1**

Step-by-step description of comparative genomics analyses with R code to explain certain steps, including discussion of results and conclusions.

**Calculating genome similarity statistics**

The *S. spontaneum* genome (query) was mapped against the *S. officinarum* genome (reference) using nucmer with default settings to produce a delta file. The show-coords script of nucmer was then run to produce an alignment coords file, which contains the start and end positions of each segment of query that can be aligned to the reference as well as the percent identity of the alignment. This file allows for duplicated overlap (for example ‘Chr1 1:10’ and ‘Chr1 3:13’ bp in the query mapping to different parts of the reference), so must be reduced to calculate unique overlap. This reduction is performed in R using the ‘reduce’ function from the package GenomicRanges to collapse overlapping ranges into a single range per reference sequence (continuing the example would collapse the range to ‘Chr1 1:13’). This reduction step then allows for the amount of query sequence to be summed ensuring that each query base is counted only once, even though it may have mapped to multiple locations. This reduction step divorces the mapped segments from the percent identity, but the average can be calculated by dividing the sum of each mapped sequence (prior to the reduction step) times its percent identity by the total sum of mapped sequence. This allows for each base to be counted only once as aligning to the reference and takes the average percent identity of each base from the multiple mapping locations that it may receive. Some important information about genome complexity can also be gathered by comparing the total mapped sequence (with duplicated overlaps) to the reduced mapped sequence. In the case of *S. spontaneum* against *S. officinarum*, the total mapped sequence was 47.61 Gb and this corresponds to only 2.88 Gb of reduced unique sequence (*S. spontaneum* genome is 3.14 Gb), meaning that there is high similarity between the homoeologous chromosomes and a large amount of repeat sequence. This high duplicate mapping is also a reflection of the size difference between the two genomes coupled with the high ploidy level. Total nucleotide identity is then calculated by taking into consideration the proportion of the query sequence that remains unmapped. Because of the way this calculation is done it can only report how much of the query matches the reference and does not describe how much of the reference matches the query, however this can be calculated by repeating the analysis and swapping the query and reference sequences.

This mapping approach was also used to compare the wheat subgenomes against each other in an attempt to shed some insight on why the hexaploid wheat genome could be phased and assembled while the KK3 genome could not. In this case we compared the subgenomes of hexaploid wheat, rather than the progenitor genomes and found that the subgenomes are less similar to each other than *S. spontaneum* is to *S. officinarum*.

**Calculating KK3 contig progenitor origins**

This analysis was intended to calculate the origin of each contig. It was performed at a time before we became aware that the assembly is chimeric and was part of the evidence for that conclusion. It has similarities to the analysis above but uses the output of BWA-MEM, specifically the CIGAR string for alignment locations, the NM tag for mismatches and insertions, and the MD tag for mismatches and deletions. We mapped the KK3 contigs (pre Hi-C) to each of the *S. officinarum* and *S. spontaneum* references to produce two sets of mapping.

bwa mem -t 10 -k 100 -B 1 -O 1 -T 500 S.offi KK3.fasta > KK3vsSoffi.sam

bwa mem -t 10 -k 100 -B 1 -O 1 -T 500 S.spon KK3.fasta > KK3vsSspon.sam

cut -f 1,3,4,6,12,13 KK3vsSoffi.sam > KK3vsSoffi_CIGAR.txt

cut -f 1,3,4,6,12,13 KK3vsSspon.sam > KK3vsSspon_CIGAR.txt

The CIGAR string files are then processed in R to calculate the start and end site of each map and the percentage similarity (accounting for mismatched bases, which are listed as mapped in the CIGAR string), and the amount of KK3 that can map to *S. officinarum* and *S. spontaneum* references were independently calculated from this. The result of this was that 4.8 Gb (68.6%) of KK3 mapped to *S. spontaneum* and 5.6 Gb (80%) of KK3 mapped to *S. officinarum*. However, there were very few contigs that mapped only to a single progenitor species. To identify progenitor ancestry we merged the reduced mapping data from both files using ‘findOverlaps’ in the GenomicRanges R package and merged the reduced files into a single file and calculated the total map and percent map per contig to each progenitor species. The resulting table matches each mapped segment overlap for each progenitor genome with the first few rows of that table shown below (Table S1), where LA is *S. officinarum* and AP is *S. spontaneum*. The colums: ‘LATotalMap’, ‘LApctMap’, ‘APTotalMap’, and ‘APpctMap’ show the total unique sequence and percent unique sequence that maps summed per contig, which can then be compared to the ‘ContigLength’. The column ‘TotalOverlap’ shows the amount of sequence of each contig that maps to both progenitor genomes and ‘PctTotalOverlap’ is that total as a percentage of the contig length.

Table S1. Mapping statistics of each contig to each progenitor genome.

Contigs LAstart LAend LAwidth LATotalMap LApctMap APstart APend APwidth

1 tig00000001 1 5962 5962 46678 0.9905776 1 1756 1756

2 tig00000001 1 5962 5962 46678 0.9905776 4133 32930 28798

3 tig00000001 6151 31352 25202 46678 0.9905776 4133 32930 28798

APTotalMap APpctMap ContigLength Overlap TotalOverlap PctTotalOverlap

1 44258 0.9392216 47122 1755 43808 0.9296719

2 44258 0.9392216 47122 1829 43808 0.9296719

3 44258 0.9392216 47122 25201 43808 0.9296719

This merged table then contains all the information needed to identify the ancestry of each contig. The assumption was that all sequence came from at least one of the progenitor species, so contigs were assigned one of five possible ancestry labels: LA, AP, equal, recombinant, unknown.

Contigs were identified as either AP or LA if they mapped to one vs the other by more than 10% (also accounting for sequence that mapped only to one and not the other).

R code: (where the table LAtoAP4 is the full version of table S1)

MostlyLAContigsList <- unique(LAtoAP4[LAtoAP4$LApctMap - 0.1 > LAtoAP4$APpctMap &

! is.na(LAtoAP4$LAstart),1])

temp <- unique(LAtoAP4[ ! is.na(LAtoAP4$APpctMap),1])

temp2 <- unique(LAtoAP4[LAtoAP4$LApctMap > 0 & is.na(LAtoAP4$APpctMap),1])

temp3 <- temp2[! temp2 %in% temp & ! is.na(temp2)]

MostlyLAContigsList <- c(MostlyLAContigsList,temp3)

rm(temp,temp2,temp3)

MostlyAPContigsList <- unique(LAtoAP4[LAtoAP4$LApctMap < LAtoAP4$APpctMap - 0.1 &

! is.na(LAtoAP4$APstart),1])

temp <- unique(LAtoAP4[ ! is.na(LAtoAP4$LApctMap),1])

temp2 <- unique(LAtoAP4[LAtoAP4$APpctMap > 0 & is.na(LAtoAP4$LApctMap),1])

temp3 <- temp2[! temp2 %in% temp & ! is.na(temp2)]

MostlyAPContigsList <- c(MostlyAPContigsList,temp3)

rm(temp,temp2,temp3)

Contigs were identified as equal if they mapped to both progenitor genomes within 10% of each other.

R code:

LAAPEqual <- unique(LAtoAP4[LAtoAP4$LApctMap - 0.1 <= LAtoAP4$APpctMap & LAtoAP4$LApctMap >= LAtoAP4$APpctMap - 0.1,1])

Recombinant contigs were defined as contigs where at least 10% of the contig aligns to one species with the remaining part(s) of the contig mapping to the other species. These contigs were identified from the contigs identified as LA or AP in the above step. The tables for this step, LARedonly and APRedonly, are entries from Table S1 that did not have an overlap in the other progenitor genome, meaning that section of the contig mapped only to a single ancestor.

R code:

LARedonly10pct <- LARedonly[LARedonly$LAwidth >= 0.1 * LARedonly$ContigLength,]

AP85Redonly10pct <- AP85Redonly[AP85Redonly$APwidth >= 0.1 * AP85Redonly$ContigLength,]

LAContigsList <- MostlyLAContigsList[! MostlyLAContigsList %in% unique(as.character(AP85Redonly10pct$seqnames)) & ! is.na(MostlyLAContigsList)]

APContigsList <- MostlyAPContigsList[! MostlyAPContigsList %in% unique(as.character(LARedonly10pct$seqnames)) & ! is.na(MostlyAPContigsList)]

RecombinantsList <- unique(sort(c(as.character(LARedonly10pct$seqnames),as.character(AP85Redonly10pct$seqnames))))[! unique(sort(c(as.character(LARedonly10pct$seqnames),as.character(AP85Redonly10pct$seqnames)))) %in% c(LAContigsList,APContigsList)]

LAAPEqualList <- LAAPEqual[! LAAPEqual %in% RecombinantsList]

length(unique(c(LAContigsList,APContigsList,RecombinantsList,LAAPEqual)))

# 104398, total contig count is 104477 so 79 contigs unknown

Since the conclusion of this work is that the long read correction step resulted in chimeric reads, it is important to understand that every step after that propagates this chimeric error. So all of the ancestry identified here carries this error. As hybridization studies have shown that the progeny inherit the full set of *S. officinarum* chromosomes and a half set of *S. spontaneum* chromosomes, this long read correction error is more likely to overwrite *S. spontaneum* sequence with *S. officinarum* sequence at regions where both progenitor genomes are more similar. However, the contigs identified here as chimeric are based on some sequence mapping only to a single ancestor and the rest of the sequence mapping to the other ancestor, so some of these are likely to be real. The number of recombinant contigs was considered abnormally high for the number of reproductive crosses that KK3 would have experienced (sugarcane is regrown from cuttings, so sexual reproduction is rare and tends to be limited to breeding new cultivars). To investigate this we performed the mapping steps described above using the R570 genome contigs against the *S. spontaneum* and *S. officinarum* genomes and identified similar mapping stats, including a high number of recombinations. The R570 genome is 530.66 Mb, 475 Mb of this can map to *S. officinarum* and 329 Mb can map to *S. spontaneum*. The comparison result showed that a total of 165 Mb mapped to both genomes with less than 10% difference and there were 407 contigs that were identified as recombinant, which is 40.7 recombinations per chromosome on average. This is fewer recombinations than the 6,819 we identified in the KK3 genome, which is 121.8 per chromosome, but both are considerably more than would be expected naturally. This is important because the R570 genome is a monoploid genome assembled using BAC inserts following gene synteny to sorghum, so it is, by design, chimeric and therefore strong evidence that the KK3 assembly is also chimeric.
