# Supplementary material for: A draft chromosome-scale genome assembly of a commercial sugarcane
Source: Sci Rep. 2022 Nov 28;12:20474. doi: 10.1038/s41598-022-24823-0 (PMC9705387; doi:10.1038/s41598-022-24823-0)
Supplement: Supplementary file 2 — Supplementary Information 2. [file 41598_2022_24823_MOESM2_ESM.pdf]

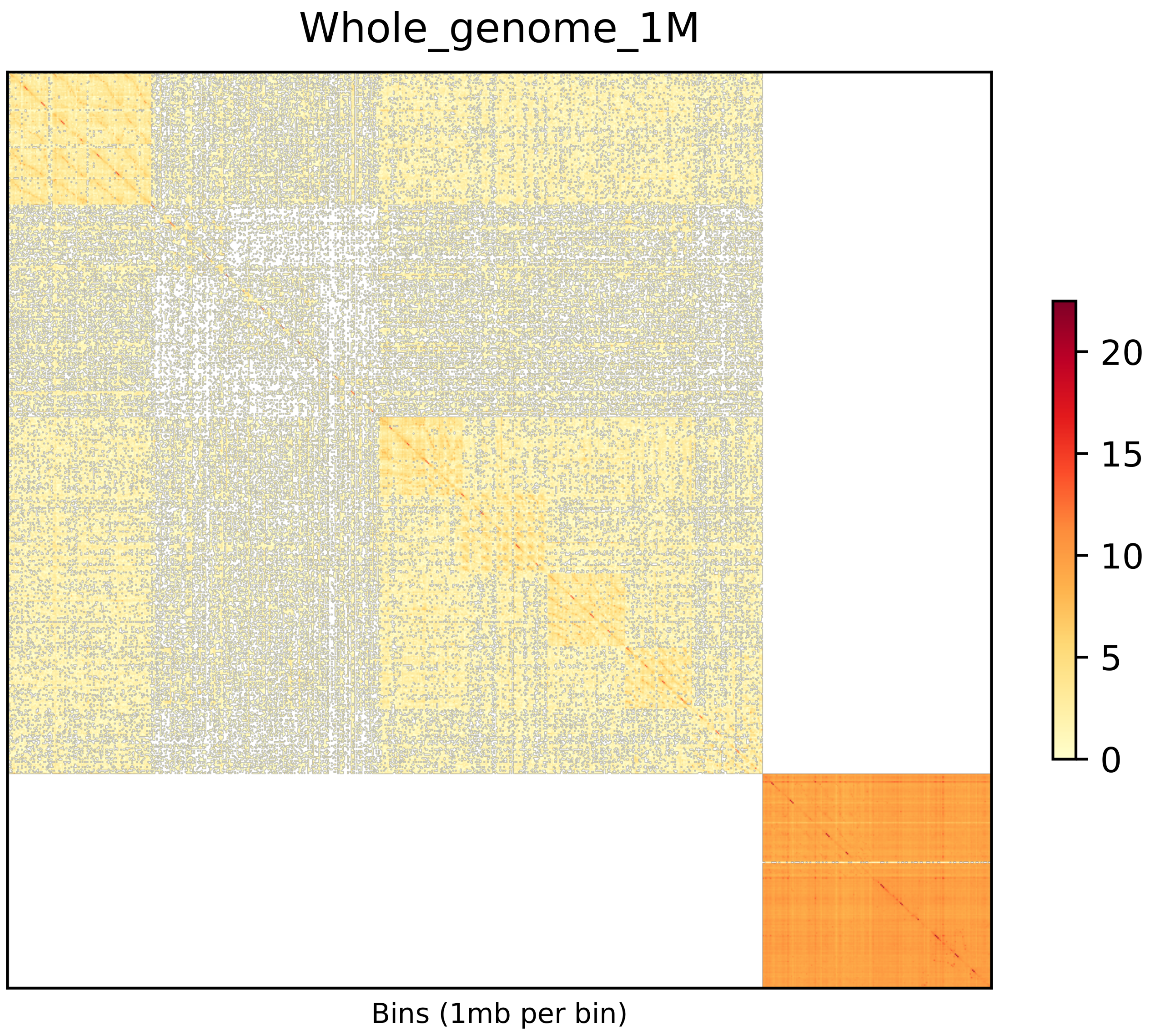

This figure is the contact map of the 56 scaffolds of KK3 plotted using 1 mb bins. The order of chromosomes from top left to bottom right is: Chr01 A,B,C,D; Chr02 B,C,D,E,F; Chr03 A,B,D,F; Chr04 B,C,E,F; Chr05 A,B,C,D,E; Chr06 A,B,C,D,E,F; Chr07 A,B,C,D,E,F; Chr08 A,B,C,D,E; Chr09 A,B,C,D,E; Chr10 A,B,C,D,E,F,G; Chr02A; Chr03E; Chr04A; Chr04D; Chr03C. The five chromosomes at the bottom right of the figure appear to form a completely separate contact group. However, there is no apparent separation of ancestral origin on a chromosome scale when considering the ancestry mapping results in this separated group. It is possible that small sections of sequence in the separated group represent sequence of *S. spontaneum* that is the most divergent from *S. officinarum* and being nested in chromosomes that were largely identified as matching *S. officinarum* is evidence that the assembly is artifactually chimeric. The low density of reads in the contact map is a reflection of how similar the homoeologous chromosomes are to each other, which results in reads mapping to many locations causing them to be discarded as multi-mapped. While the contact density shows a severe lack of evidence supporting the contig order in many of the chromosomes, the gene synteny analysis shows that the order of the contigs that contain genes is correct.
