# Supplementary figures and images for: A draft chromosome-scale genome assembly of a commercial sugarcane
Source: Sci Rep. 2022 Nov 28;12:20474. doi: 10.1038/s41598-022-24823-0 (PMC9705387; doi:10.1038/s41598-022-24823-0)

Repeat masked KK3 Contigs that map to spontaneum or officinarum

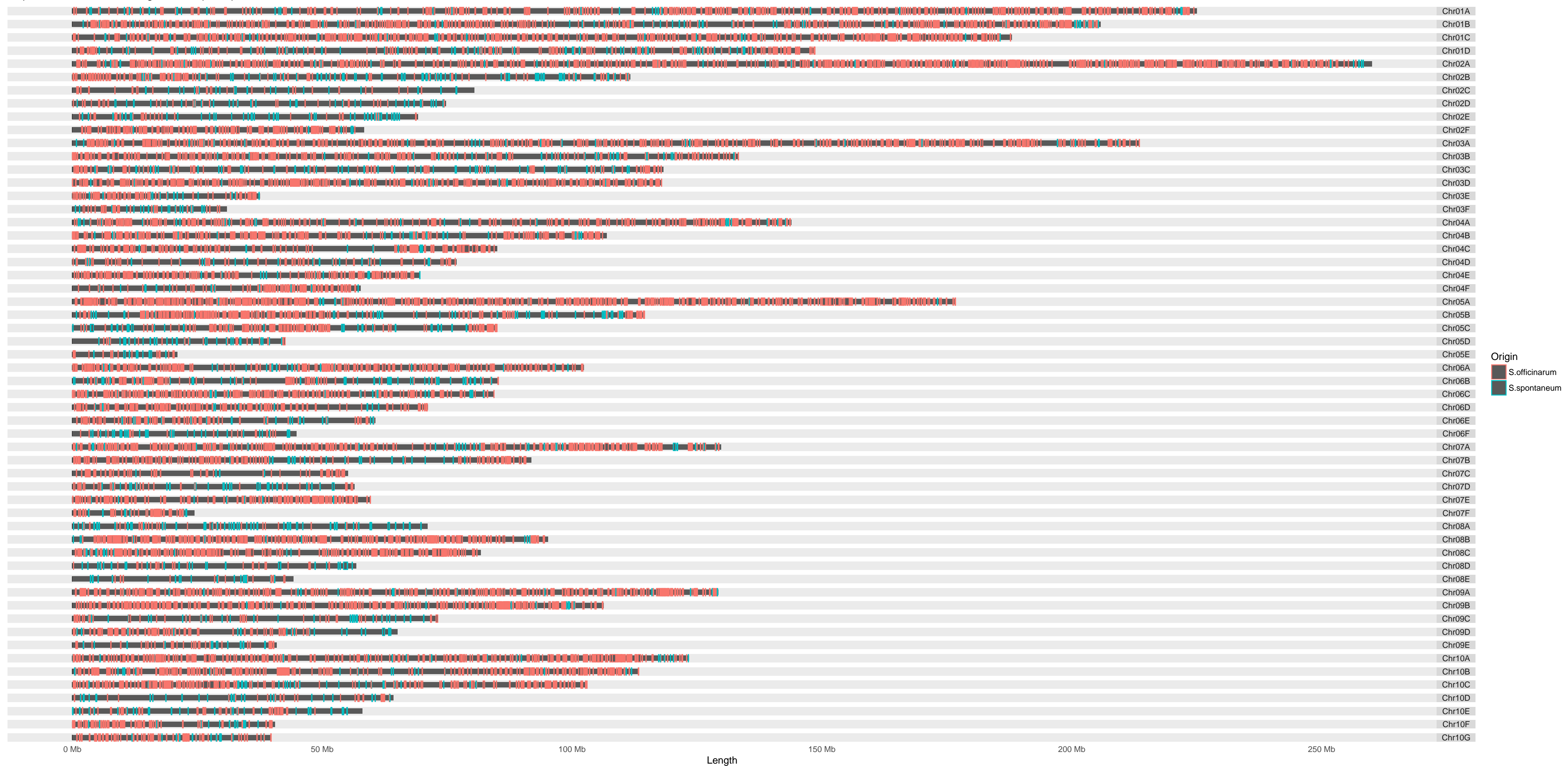

Supplement: Supplementary file 3 — Supplementary Figure 1. [file 41598_2022_24823_MOESM3_ESM.pdf]

Inter-genomic comparison: KK3Sspont vs Sb (15871 gene pairs)

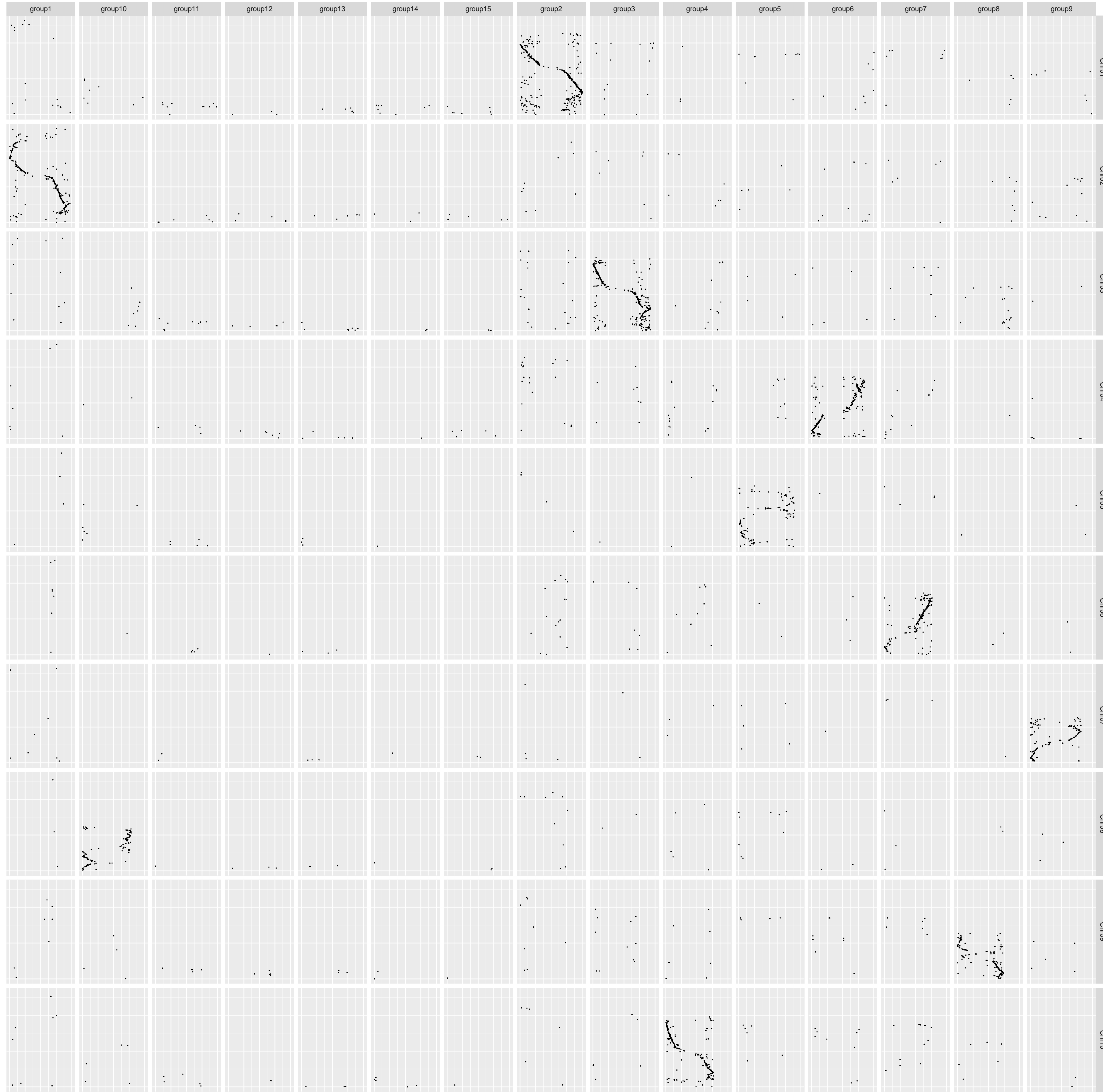

KK3

Sb

Supplement: Supplementary file 4 — Supplementary Figure 2. [file 41598_2022_24823_MOESM4_ESM.pdf]
